# Internal Medicine Residents and the Practice of Defensive Medicine: A Pilot Study Across Three Internal Medicine Residency Programs

**DOI:** 10.7759/cureus.6876

**Published:** 2020-02-04

**Authors:** Saif M Borgan, Laniel Romeus, Saleh Rahman, Abdo Asmar

**Affiliations:** 1 Internal Medicine, University of Central Florida College of Medicine, Orlando, USA; 2 Epidemiology and Public Health, University of Central Florida College of Medicine, Orlando, USA

**Keywords:** defensive medicine, resident training, internal medicine resident, resident education, healthcare spending, malpractice, medical education, medico-legal, medical malpractice

## Abstract

Background

Defensive medicine is becoming increasingly prevalent in the United States and is estimated to cost billions of dollars in excess healthcare spending. There is evidence that the practice of defensive medicine starts early in the medical career. Defensive medicine has been investigated among residents in high medico-legal risk specialties, but there is a paucity of information on its prevalence among internal medicine residents.

Objective

To examine the prevalence and patterns of defensive medical practices among internal medicine residents.

Methods

We conducted an online survey among the residents of three internal medicine residency programs in the 2018-2019 academic cycle. We invited all internal medicine residents within the selected programs to participate through email and asked them to complete an electronic survey assessing defensive medical practices.

Results

A total of 49 out of 143 residents participated in the study (response rate: 34.3%); 55% (n = 27) of the residents who participated considered the risk of being sued during residency to be low, compared to 40.8% (n = 20) who considered it to be moderate and 4.1% (n = 2) who considered it to be high. Defensive medical practices were found to be widely prevalent (40.0-91.3%) among internal medicine residents across all three clinical training stages. Assurance defensive practices were more common than avoidance practices.

Conclusion

Defensive medical practices, especially of the assurance type, were widely prevalent among our sample of internal medicine residents.

## Introduction

Defensive medicine is the deviation from routine medical care in order to avoid or reduce the risk of real or perceived future legal consequences [1-2]. Defensive practices are categorized into two distinct patterns: assurance practices that unnecessarily over-investigate lower-risk patients, and avoidance practices that aim to avoid intervention in the care of higher-risk patients [3]. The practice of defensive medicine can put patients at significant risk and may also involve a significant economic burden [2]. A study in 1984 estimated the national cost of defensive medicine to be 37 billion dollars per year, constituting 14% of the total healthcare costs [4]. Another study published in 1994 estimated the excess cost of defensive medicine to be over 41 billion dollars in a five-year period [5]. One more recent study in 2004 judged 28% of physician orders and 13% of costs to be at least partially defensive in nature [6]. This comes at a time when the US government is struggling to keep up with steep healthcare costs. There is increasing evidence of defensive practices among physicians in medical and surgical specialties [7-8]. However, defensive practices among internal medicine residents remain largely unknown [9]. O’Leary et al. published a study in 2012 comparing defensive medicine practices among third-year medical students and third-year residents in one institution among various specialties. The study found that both medical students and residents were often engaging in assurance practices. In addition, they were being taught to take malpractice liability into consideration during clinical decision making [10]. Another longitudinal study of emergency medicine residents published in 2007 concluded there were no significant differences in malpractice liability concerns or defensive medicine between interns and graduates across a four-year period [11]. Brilla et al. conducted a comparative study in 2006 between neurology residents in the US and Germany and concluded that US residents are more likely to consider medical liability as a problem and more likely to practice defensive medicine [12]. There are no studies investigating defensive medicine specifically among internal medicine residents. Based on anecdotal reports, we hypothesized that defensive medical practices would be widely prevalent among internal medicine residents. We conducted a cross-sectional study to investigate self-reported defensive medical practices and their patterns among internal medicine residents.

## Materials and methods

Study population and settings

We conducted an online, survey-based study assessing defensive medicine practices. Residents across three internal medicine residency programs in Central and North Florida were invited to participate. The three programs were chosen based on convenience (programs operated under one regional university affiliation), and they had a capacity of 68, 45, and 30 residents, respectively. All three programs can be described as university-affiliated with the main teaching site being a community regional medical center for each. In the largest participating program, residents spent approximately 50% of their clinical duties in a Veterans Affairs (VA) hospital. Six months into the academic year of 2018-2019 (November 2018), emails were sent inviting residents to participate by completing an online questionnaire on defensive medicine. We sent reminder emails to survey non-responders every two weeks for six weeks (a total of three reminders).

Questionnaire

A previously created questionnaire by Studdert et al. was adapted for use in this study [3]. The original survey was used to examine the prevalence of defensive medicine among “high risk” specialties (emergency medicine, general surgery, orthopedic surgery, neurosurgery, obstetrics/gynecology, and radiology) and developed through interviews with representatives in the medical community, societies, insurers, and hospital and government agencies. In the current study, we amended the questionnaire to suit the resident population. Face and content validity were done by receiving expert opinions and feedback from residents and their clinical educators. The reliability of the instrument is yet to be determined with a larger sample size. The survey included closed-ended Likert scale questions assessing the frequency of various defensive medicine practices of both assurance and avoidance types. Additional questions included those relating to assessed baseline medico-legal risk perception, taught defensive medicine, and effect on career satisfaction. The medico-legal risk was defined as being named in a lawsuit, regardless of the lawsuit outcome. The questionnaire is accessible as supplementary data.

Ethical considerations

We obtained approval from the University of Central Florida Institutional Review Board (approval number: SBE-18-14072). An informational sheet about the study was emailed to each resident. No information of a personal or identifiable nature was obtained to protect the anonymity of the respondents.

Statistical analysis

Survey responses were analyzed using the Statistical Package for the Social Sciences (SPSS) software version 22.0 (IBM, Armonk, NY). The quantitative data were presented in figures and tables. We performed Chi-square tests of independence, and a p-value of ≤0.05 was considered statistically significant.

## Results

A total of 49 out of 143 residents participated in the study (response rate: 34.3%). Response rates within each of the participating programs were 16.7%, 20%, and 50% respectively. The baseline characteristics of the participants are presented in Table 1. All but one resident (n = 48, 98%) denied being personally named in a lawsuit or knowing any colleague who had been named.

**Table 1:** Baseline characteristics of internal medicine resident respondents across three training programs (total respondents = 49)

Characteristic	Number	Percent (%)
Age, years		
25-27	9	18.8
28-29	13	27.1
30-32	16	33.3
33-35	4	8.3
>36	6	12.5
Gender		
Male	31	66.0
Female	16	34.0
Ethnicity		
White	25	51.0
Black	2	4.1
Hispanic	11	22.4
Asian	7	14.3
Other	4	8.2
Level in residency		
Postgraduate year 1	18	36.7
Postgraduate year 2	22	44.9
Postgraduate year 3	9	18.4
Graduation country		
International graduate	44	89.8
United States graduate	5	10.2
Clinical experience prior to residency		
No	33	67.3
Yes	16	32.7

Defensive medical practices and the role of supervision

**Figure 1:**
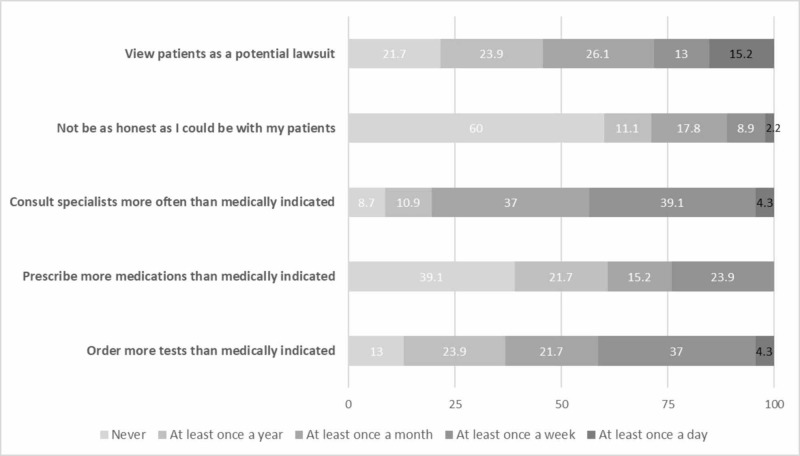
Self-reported frequency of defensive medical practices among internal medicine residents surveyed (total respondents = 49)

The details of defensive medical practices obtained from the survey are presented in Figure 1. There were no statistically significant differences in defensive practices between residents with prior clinical experience and those without. The details of defensive practices within each of the clinical training years are presented in Table 2.

**Table 2:** Frequency of defensive medical practices among survey respondents by training level and experience (total respondents = 49)

	Training year 1		Training year 2		Training year 3		P-value
	Number of residents	Percent (%)	Number of residents	Percent (%)	Number of residents	Percent (%)	
Prior clinical training							0.16
No	15	83.3	12	54.5	6	66.7	
Yes	3	16.7	10	45.5	3	33.3	
Graduation country							0.09
International	15	83.3	33	100.0	7	77.8	
United States	3	16.7	0	0.0	2	22.2	
Perceived risk of malpractice lawsuit during residency							0.19
Low	7	38.9	12	54.5	8	88.9	
Moderate	10	55.6	9	40.9	1	11.1	
High	1	5.6	1	4.5	0	0.0	
I view patients as a potential lawsuit							0.23
Never	6	35.3	3	15.0	1	11.1	
All other responses	11	64.7	17	85.0	8	88.9	
I am not as honest as I could be with my patients							0.19
Never	10	58.8	10	50.0	7	87.5	
All other responses	7	41.2	10	50.0	1	12.5	
I consult specialists more often than clinically indicated							0.58
Never	2	11.8	2	10.0	0	0.0	
All other responses	15	88.2	18	90.0	9	100.0	
I prescribe more medication than clinically indicated							0.42
Never	7	41.2	6	30.0	5	55.6	
All other responses	10	58.8	14	70.0	4	44.4	
I order more tests than medically indicated							0.94
Never	2	11.8	2	15.0	1	11.1	
All other responses	15	88.2	17	85.0	8	88.9	

When asked whether they were being explicitly told by attending physician to consider medico-legal risk prior to a medical decision, only five residents answered “never” (10.2%) compared to nine residents who answered “at least once a year” (18.4%), 18 residents who reported “at least once a month” (36.7%), 13 reporting “at least once a week” (26.5%), and four who answered “at least once a day” (8.2%). As for peer-to-peer defensive medicine education (senior resident to junior resident), 13 residents reported “never” (27.1%) compared to 9 who reported “at least once a year” (18.8%), 11 reporting “at least once a month” (22.9%), 13 who reported “at least once a week” (27.1%), and 2 who reported “at least once a day” (4.2%).

Medico-legal risk perception 

**Figure 2:**
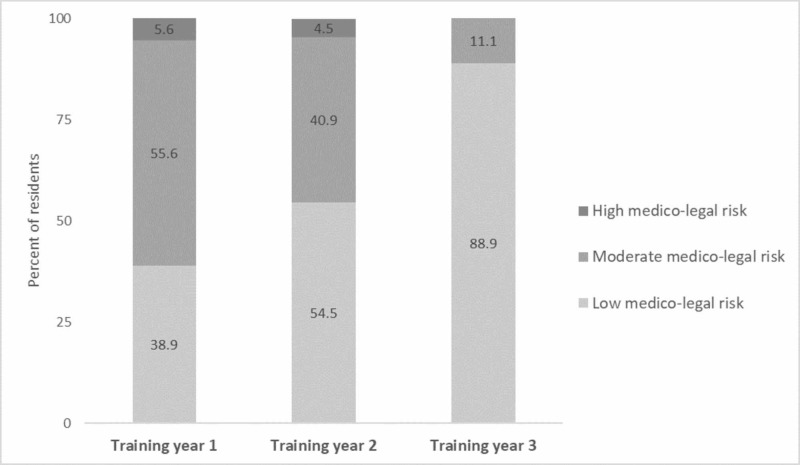
Internal medicine residents' perception of medico-legal risk by training level (total respondents = 49)

Twenty-seven residents (55.1%) considered their medico-legal risk during residency to be low compared to 20 residents (40.8%) who considered it to be moderate and two residents (4.1%) who considered it to be high. There was a clear trend towards a perception of lower risk with the increase in training levels as shown in Figure 2. However, this was not statistically significant (p: 0.19 and 0.20, respectively). 

Impact on future career and satisfaction

Forty-two residents reported that concerns about medico-legal risk negatively impacted their career satisfaction (85.7%) compared to seven residents who reported no negative impact (14.3%). Among those who reported negative impact, 16 residents reported “slight negative impact” (32.7%), 19 residents reported “somewhat negative impact” (38.8%), and seven residents reported “very negative impact” (14.3%). There was no difference in career impact between postgraduate year-1 (PGY-1) students and more senior residents (PGY-2 and PGY-3). Forty-six residents reported they would consider medico-legal risk when making medical decisions in their future practice (93.9%) compared to three residents who would not consider it (6.1%).

## Discussion

In an increasingly litigious society, defensive medicine is ever more prevalent [7-11]. The increasing practice of defensive medicine increases healthcare spending by billions of dollars annually as suggested by studies conducted as early as the 19th century [5]. Evidence suggests defensive medicine practices begin during early career development, before specialty training, even as early as during medical school [10]. Studies aiming to explore defensive medical practices are necessary to understand and counteract this phenomenon. In this study, we showed that defensive medicine practices were widely prevalent among our sample of internal medicine residents across all three clinical training stages. A considerable portion of residents reported their medico-legal risk during residency to be moderate, and a majority of them felt that concerns about medico-legal risk negatively impacted their career satisfaction.

There is some evidence that the true risk of medical malpractice claims among physicians is overstated [3]. For example, one study found that only 10% of malpractice claims actually went to trial, with a majority either dropped or settled out of court [3]. For those that progress to trial, the verdict favors healthcare providers 80% of the time [3]. In our study, 44.9% of residents did not consider their medico-legal risk to be low. The reasons behind this discrepancy between actual and perceived medico-legal risk should be explored further. There was a trend towards lower risk perception with the increase in training levels; however, this trend was not statistically significant. Future studies with a larger sample size may explore this further. 

Assurance defensive practices (consulting specialists unnecessarily and ordering more tests) were more prevalent than avoidance practices such as not being honest with patients. This is consistent with other studies investigating physicians in training [10]. This pattern may be explained by the perception of risk to patients. While consulting a specialist does increase healthcare spending, it does not necessarily put patients at health risk or interfere with the physician-patient relationship. However, this perception exposes the obvious disconnect between cost consideration and medical decision making among modern physicians. For example, in one cross-sectional electronic survey study among physicians from the American Medical Association (AMA) Masterfile, only 36% felt they had a “major responsibility” in reducing healthcare costs [13].

In one cross-sectional study conducted among third-year medical students in the United States, students were rarely concerned about medical lawsuits [14]; however, 55.9% felt their faculty were concerned about it, and 32.4% reported faculty actively teaching them defensive medicine practices [15]. The results of our study are consistent with those findings and with findings across the United States. In our study, resident defensive medicine teaching by seniors (PGY-2, PGY-3) to juniors (PGY-1) is also reported; however, being taught defensive medicine by an attending physician or a resident was not correlated with a higher frequency of defensive medicine practices in the current study.

Career satisfaction is particularly important in medicine because it is linked to the quality of care [15]. There is evidence that career dissatisfaction and burnout are increasing in the field of medicine [16]. Medico-legal risk perception in medicine may be a contributing factor. In our study, the majority of the residents reported at least some negative impact from defensive medicine on their career satisfaction.

Tackling defensive medicine will require a multi-system approach. Defensive medicine is widely prevalent in the United States across different specialties, varying training levels, and major academic and non-academic healthcare settings. The full impact of defensive medicine should also be further explored and monitored on both institutional and national levels.

We believe that minimizing defensive practices requires comprehension of its driving forces across the patient population, healthcare professionals, healthcare institutions, media, legal systems, and national policies [17]. The patient-physician relationship is deteriorating in modern medicine [17]. Decreasing face-to-face time in clinical encounters, a decline of thorough physical exams, and the ease of patient access to contradictory electronic medical information and second opinions have led to a gap in patient-physician trust. Additionally, the over-reliance on guidelines and algorithms has minimized the personal nature of medical practice, replacing it with assembly-line medicine [17]. Given that medico-legal claims favor errors of omission the most, positive defensive medical practices are further incentivized [16]. This phenomenon is also driven by poor physician knowledge and clinical reasoning and the need to “rule out” various diseases [18]. Further, the lack of cost-consciousness among modern physicians needs to be addressed, preferably with a mandatory national residency curriculum as well as local institutional orientation. Interestingly, healthcare institutions may find defensive medicine profitable as reimbursement systems often reward appropriate and inappropriate use of medical testing [18]. The exaggerated media coverage of malpractice lawsuits and the promotion of the profitable industry of malpractice claims continue to drive a wedge between the general population and the physician [16]. From a national policy standpoint, decriminalization of medical errors and reducing the profitability of medico-legal claims may assist in reducing defensive medicine practices. Residents in training are particularly susceptible to learning defensive medicine practices. The graduate medical education community must see this as an opportunity for early intervention by implementing educational strategies targeting both faculty and residents. Practicing medicine is built upon dealing with clinical uncertainties and cannot always be addressed by using “rule out” or over-testing strategies. Further studies are needed to explore and develop strategies to move medicine towards a benchmark of less testing and more communication.

The limitations of our study included selection, non-respondents, and observer biases. Our sample size was limited to residents participating in three programs in one state. These programs have a high percentage of international medical graduates. This may reflect a different level of defensive medical practices not generalizable to the entire US resident pool. Since the malpractice environment varies by state, our population may be more or less inclined to practice defensive medicine than other comparable resident populations [19]. A nationwide study about defensive medicine practices would potentially address this issue. Additionally, since we administered a self-reported questionnaire, we were subject to observer and non-respondents’ biases. However, the online distribution of the questionnaire may have minimized these biases.

## Conclusions

Defensive medical practices, especially of the assurance type, were found to be prevalent among our sample of internal medicine residents. A significant proportion of residents considered their medico-legal risk during residency to be moderate. Nationwide studies are required to further understand and quantify this phenomenon among internal medicine residents across the United States.

## Appendices

Questionnaire 

Q1. Have you or any of your colleagues ever been named in a malpractice suit?

o  No, Never

o  Yes

Q2. In your opinion, your risk of malpractice lawsuit during residency is:

o Low 

o Moderate 

o High 

Q3. How often do concerns about malpractice lawsuits cause you to

-Order  tests that are not medically indicated 

o Never 

o At least once a year 

o At least once a month 

o At least once a week 

o At least once a day

-Prescribe  medications that are not medically indicated

o Never

o At least once a year

o At least once a month

o At least once a week

o At least once a day

-Consult specialists more often than needed

o Never

o At least once a year

o At least once a month

o At least once a week

o At least once a day

-View patients as a potential lawsuit

o Never

o At least once a year

o At least once a month

o At least once a week

o At least once a day

-Not be as honest as I could be with patients

o Never

o At least once a year

o At least once a month

o At least once a week

o At least once a day

Q4. What impact has concerns of malpractice lawsuits had on your career satisfaction?

o No negative impact at all

o Slight negative impact

o Somewhat negative impact

o Very negative impact

Q5. How often does your attending physician explicitly recommend that you consider lawsuit risk when you make clinical decisions in the future?

o Never

o At least once a year

o At least once a month

o At least once a week

o At least once a day

Q6. During your intern year, how often did your supervising resident explicitly recommend that you consider lawsuit risk when you make clinical decisions in the future?

o Never

o At least once a year

o At least once a month

o At least once a week

o At least once a day

Q7. Do you plan to consider malpractice lawsuit risk when making medical decisions in your future practice?

o No

o Yes

Q8. Specify age group

o Less than 27 years old

o 28-30 years old

o 31-35 years old

o 36 years old or more

Q9. What is your sex?

o Male

o Female

o Other ________________________________________________

o Prefer not to disclose

Q10. Which ethnicity do you most identify with?

o White

o Black

o Hispanic or Latino

o Asian

o Other

Q11. Are you an international medical graduate?

o Yes

o No

Q12. Year of residency training

o PGY-1

o PGY-2

o PGY-3

Q13. Location of residency training

o Residency program 1

o Residency program 2 

o Residency program 3

Q14. Have you completed any formal clinical training prior to beginning residency (ie, residency in another country)?

o Yes

o No
